# Dementia-Related Care in Acute Care Settings: A Qualitative Meta-Synthesis of Patient and Family Perspectives

**DOI:** 10.1093/geroni/igab046.3584

**Published:** 2021-12-17

**Authors:** Richard Chunga

**Affiliations:** University of Massachusetts Boston, Kew Gardens, New York, United States

## Abstract

Researchers have long emphasized the importance of a person-centered approach to health care, especially regarding the treatment of individuals living with dementia. However, the fast pace of acute care settings can be a difficult place to provide such care to patients, where there are tensions between the emphasis on efficient treatment of acute medical co-morbidities and person-centered dementia care. This paper is a meta-synthesis of qualitative studies examining perspectives of patients and their family members regarding their acute care experiences. It takes an interpretive approach, using primarily inductive reasoning to generate themes across available studies’ findings. Emergent themes are organized under two major dimensions of the hospital environment: the physical environment, including sensory and tangible elements, and the social environment, including the hospital atmosphere and communication practices. Persons with dementia feel overly stimulated by the busy physical environment of the hospital, yet they are often left to languish alone, sometimes even physically restrained. Patients reported feeling lonely, fearful, and confused, identifying diverse physical and social environmental attributes like physical clutter, noise, and lack of empathy from care providers as contributors. Based on acute care experiences and reports from patients and family members, although the acute condition is treated, persons with dementia often leave the acute care environment in worse functional condition than when they entered. Given the increasing prevalence of persons with dementia in acute care settings, it is clear that we must prioritize innovations and programs aimed at improving hospital practices, educating staff, and creating more dementia-friendly environmental designs.

